# Supplementary material for: Learning curve and surgical outcome of robotic assisted colorectal surgery with ERAS program
Source: Sci Rep. 2022 Nov 29;12:20566. doi: 10.1038/s41598-022-24665-w (PMC9709162; doi:10.1038/s41598-022-24665-w)
Supplement: Supplementary file 1 — Supplementary Information 1. [file 41598_2022_24665_MOESM1_ESM.zip › LSC Raw data-Ñ[▒K/120/H_ñw╝╨░O▒Kñσ.pdf]

正式摘要(電子簽章：[illegible])
W55 / W55 ☐ 英文標題(注意:無英文姓名-請補病人基本檔!) ☐【診取】憑證

臺中榮民總醫院

出院病歷摘要

轉入號： 轉出號：

[redacted]

(8)流水編號	
(9)入院日期	110/03/03 (2021) 直腸外科 W55 - 112
(10)轉科日期	
(11)出院日期	110/03/30 (2021) 住院天數計 27 日

診斷

- 入院：Sigmiod colon cancer, cT3 N0, stage IIA
  <Hx>
  2. HTN
  3. COPD
- 出院：Sigmiod colon cancer, cT3 N0, stage IIA, s/p Laparoscopic low anterior resection w/ high ligation (IMA root, IMV, left colic artery) on 2021/03/12
  Patho: Tubulovillous adenoma with focal high grade dysplasia
  <Hx>
  2. HTN
  3. COPD

主訴

- admitted for nutrition support

病史

- This 77 y/o male with a hx of
  1. Sigmiod colon cancer, c T3 N0, Stage IIA
  2. HTN
  3. COPD
  In 2020, he complained constipation for 2 months. Body weight loss about 20kg in 1 year was also noted. He first went to LMD where colonoscopy was done and showed a S-colon tumor. Colonoscopy on 12/14 showed a tumor over 30cm FAV. Due to large tumor size and partial obstruction, operation was suggested. However, nutrition support was suggested but the patient hesitated and decided to AAD on 2020/12/28 even risks were informed. This time, under the impression of Sigmiod colon cancer, c T3 N0, Stage IIA, he was admitted for nutrition support and operation if indicated.

體檢發現

- NUTRITION AND SUPPORTIVE TPN BEFORE OP...................
  - General appearance :
    - easy looking , conscious clear , without cardiopulmonary distress
    - Vital signs自動帶入date 體溫(BT) 脈搏(P) 呼吸(R) 血壓(BP) 身高(BH) 體重(BW) BMI
      20210303 35℃ 79次/分 18次/分 154/91mmHg 166.6cm 42.4kg 15.3
    - Vital signs: B.P. 154/91 mmHg , P.R. 79 /min , R.R. 18 /min , B.T. 35 ℃
  - HEENT :
    - Eyes: no anemic conjunctiva, no icteric sclera, normal EOM
    - ENT: no otorrhea, no rhinorrhea, no erythematous mucosa, no injected tonsil, no deformity
  - Neck: supple, freely movable, trachea midline,no deformity, no engorged jugular vein , no palpable thyroid or lymph node enlargement
  - Chest:
    - Inspection: Grossly normal, symmetric and fully expansion, no accessory muscle used, no operation scar, no spider angiomata, no gynecomastia
    - Palpation: no palpable mass or nodule, no subcutaneous emphysema, no tenderness
    - Ausculation: clear breathing sound
  - Heart:
    - Palpation: no thrill, no heave
    - Percussion: normal heart size
    - Ausculation: regular heart beat, normal S1, S2, no gallop, no murmur
  - Extremities: No deformity, full ranged of motion without limitation, no clubbing finger or nail cyanosis, no pitting edema, no ulcer or pigmentation, no obvious skin lesion, no varicose vein, no bone, joint or muscle tenderness

  CRS CONDITION
  - Karnofsky performance status: (100) Normal, no complaints, no evidence of disease
  - PE of Abdomen:
  - CEA: 1.3 ng/ml
  - Imaging Studies:
  - Colonoscope report: showed a tumor over 30cm FAV
  - Figures:
    1. colon & rectum

手術日期及方法

- 手術日期及方法20210312 1. Laparoscopic low anterior resection was done
  2. Intraoperative colonoscopy : (-)
  3. High ligation (+) : At IMA root. IMV and left colic artery were also ligated.
  4. EndoGIA COVIDIEN ENDO GIA 60mm (Medium Thick) X2 + EEA (29 mm) were applied.
  5. Proximal cut end: 15 cm, distal cut end: 2 cm
  6. The colon was not taken down from the splenic flexure site

住院治療經過

- After admission, we consulted TPN for pre-op nutririon support. We also cinsuted CCRT, SICU,REH for pre-op and post-op care. Better spirit and appetite were noted these days. Laparoscopic low anterior resection w/ high ligation (IMA root, IMV, left colic artery) were done smoothly on 2021/3/12 . He was then trasferred to SICU after op. At SICU, for his poor lung condition, we kept sedation use for five days. We tried water then diet since 3/13. Kinking NG was also noted, we changed the new one. The patient woke up on 3/17 but he was irritable and hard to cooperate ventilator. He was transfered to RICU due to AFS postive, but TB-PCR revealed negative. ETT was removed ob 03/24. He still needed BiPAP for support due to COPD. Under relative stable condition, he was transferred to ordinart ward on 2021/03/26. We removed stitches on 03/29 then discharged the patient on 03/30.

合併症

一般檢查

- 累積報告：

DATE	TIME	ALB	TP	BIL.T	BIL.D	ALKP	AST	ALT	LDH	CHOL	TG	HDL-C	LDL	UA	NH3	AMY
1100303	1902	3.5	8.2	0.62	0.12	126	27	10	296							
1100304	0916									108	47					
1100312	1455			1.19				12								
1100313	0547			0.90	0.24		12	10								
1100314	0550	2.2		0.70	0.21		14	7								
1100315	0517			0.98	0.26		14	5			47					
1100317	0521			1.26	0.45		30	9	137							
1100319	0500			0.91	0.33		24	8	176							
1100321	0510	3.0		0.84	0.28		14	6			74					
1100322	0517			0.8	0.3		18	8								

1100322	2133			0.45	0.17											
1100325	0503			1.03	0.29											

DATE	TIME	WBC	RBC	HGB	HCT	MCV	PLT	BLST	NEUT	BAND	SEG	LYM	MON	EOS	BAS
1100303	1902	6050	4.31	8.8	31.2	72.4	306		61.3			30.6	6.1	0.8	1.2
1100308	0924	6320	3.95	8.4	28.9	73.2	281		41.7			47.5	5.7	4.0	1.1
1100311	1204			8.8	28.5										
1100312	1456	8730	3.69	9.3	28.4	77.0	182		88.1			8.2	3.3	0.1	0.3
1100313	0542	6320	3.84	9.0	29.4	76.6	177		77.4			15.3	4.6	2.2	0.5
1100314	0547	6600		9.0	27.8	78.3	163		74.1			15.8	5.6	3.9	0.6
1100315	0508	6920	3.50	8.4	27.5	78.6	141		73.9			17.6	4.2	3.9	0.4
1100316	0636			8.7	29.4										
1100317	0521	9020		9.0	30.1		134		81.3			10.1	4.2	4.1	0.3
1100319	0458	6970	3.64	9.0	28.9	79.4	161		79.0			13.6	4.4	2.7	0.3
1100321	0509	8060	3.60	8.8	29.1	80.8	211		67.9			22.0	4.3	5.1	0.7
1100322	0512	5960	3.42	8.5	27.7	81.0	233		63.3			22.8	3.7	9.4	0.8
1100322	2131	5230	3.47	8.5	28.2	81.3	269		68.2			17.4	3.4	9.9	1.1
1100325	0503	9530	4.21	10.5	34.7	82.4	467		74.1			17.8	3.9	2.8	1.4

DATE	TIME	NA	K	CL	CA	BUN	CREAT	Mg	P	CRP	ESR	PCT	LIPASE	LACTATE
1100303	1902	140	4.7	101	8.6	14	0.91	2.7	4.2					
1100308	0911	140	4.4			11	0.77							
1100312	1455	141	3.0			12	0.73							
1100313	0543													12.9
1100313	0547	143	2.7	106	7.6	14	0.77							
1100314	0550	143	3.0	109	7.3	14	0.75							
1100315	0509													6.9
1100315	0517	144	3.2	112	7.1	12	0.68	1.8	3.4					
1100316	0635	141	3.4											
1100317	0518													8.0
1100317	0521	141	3.6	107	7.5	17	0.75							
1100319	0458													10.1
1100319	0500	144	3.5	105	8.0	27	0.72							
1100320	0606		4.4											
1100321	0510	142	3.7		7.9	32	0.92							
1100322	0515													9.9
1100322	0517	142	3.5	103	7.7	28	0.82							
1100322	2131													11.0
1100322	2133	140	4.2				0.77							
1100325	0503	141	4.2				0.89							

DATE	TIME	GLUCOSE	PAB	TRF	ZN	CU	KETONE	CK	CKMB	PT-P	PT-C	APTT-P	APTT-C	TRO-T
1100303	1901									10.8	10.6	24.8		
1100312	1455	148												

DATE	TIME	SP-GR	PH	PROT	GLUC	KETON	BILI	UROB	OB	NITR	LEU	RBC	WBC	EPCEL
1100312	1917	1.039	6.0	2+	Negative	Negative	Negative	0.2	3+	Negative	1+	2+	5-10	0-1

DATE	TIME	STOOL-OB	FAT	RBC	WBC	PARA
1100322	2139	2+				

DATE	TIME	PH	PCO2	PO2	SO2	BEB	HCO3	TCO2	HGB	FIO2
1100313	0548	7.437	42.5	94.4	97.7	3.4	28.1	29.4		
1100315	0509	7.403	41.6	115.6	98.5	0.5	25.4	26.6		
1100319	0505	7.478	43.6	99.2	98.3	7.2	31.7	33.0		
1100322	2127	7.449	43.8	102.6	98.2	5.0	29.7	31.1		
1100324	1407	7.416	50.4	59.1	91.1	5.7	31.6	33.2		
1100325	0502	7.463	37.6	57.1	91.3	2.7	26.4	27.5		

DATE	TIME	PH	BEB	SCVO2	PO2	HCO3	HGB	PCO2	TCO2
1100312	1453	7.433	2.6	99.5	164.4	27.2		41.6	28.5

- 放射線報告 :檢驗項目 **:CHEST AP VIEW (PORTABLE)** 報告日期 **:1100322**

主 訴：
Result:N
Finding:
> The heart size is within normal limit
> Calcification of thoracic aorta
> Bilateral hilar prominence
> Increased insterstitial infiltration with haziness over bilateral lower lung fields
> Fibrotic change over bil. apical lung regions with patchy opacities at bilateral upper lung fields, chronic granulomatous disease should be considered, suggest clinical correlation and follow up.
> Mild blunting of left CP angle, may be due to pleural effusion or pleural change
> Degenerative change of thoracic and lumbar spine with osteophytes formation.
> Old fracture of right ribs and right clavicle
> On endotracheal tube, nasogastric tube and EKG leads
Impression:
As above description.

檢驗項目 **:CHEST AP VIEW (PORTABLE)** 報告日期 **:1100326**
主 訴：
Result:N
Finding:
> The heart size is within normal limit
> Calcification of thoracic aorta
> Patches of hazy opacities over bilateral lung fields are noted. Infective process could be considered. Suggest clinical correlation and F/U.
> Fibrocalcified lesions in bilateral upper lung fields. Previous granulomatous infection should be considered.
> Mild blunting of bilateral CP angle, may be due to pleural effusion or pleural change
> Degenerative change of thoracic and lumbar spine with osteophytes formation.
> Old fracture of right ribs and right clavicle
> On endotracheal tube, central venous catheter, nasogastric tube and EKG leads
Impression:
As above description.

檢驗項目 **:CHEST AP VIEW (PORTABLE)** 報告日期 **:1100319**
主 訴：
Result:N
Finding:
> The heart size is within normal limit
> Calcification of thoracic aorta
> Left hilar prominence.
> Patchy opacities and alveolar infiltration over bilateral upper lung fields are noted. Infective process could be compatible. Tumor growth can't be excluded, suggest further evaluation.
> Left pleural effusion.
> Degenerative change of thoracic and lumbar spine with osteophytes formation.
> Old fracture of right ribs and right clavicle
> On endotracheal tube, central venous catheter, nasogastric tube and EKG leads
Impression:
As above description.

檢驗項目 **:CHEST AP VIEW (PORTABLE)** 報告日期 **:1100314**
主 訴：
Result:N
Finding:
> The heart size is within normal limit
> Calcification of thoracic aorta

- > Patches of alveolar infiltration over bilateral upper and right lower lung fields are noted. Infective process could be compatible. Tumor growth can't be excluded, suggest further evaluation.
  > Mild blunting of bilateral CP angle, may be due to pleural effusion or pleural change
  > Degenerative change of thoracic and lumbar spine with osteophytes formation.
  > Old fracture of right ribs and right clavicle
  > On endotracheal tube, central venous catheter and EKG leads
  Impression:
  As above description.

特殊檢查

病理報告

- 活體組織切片病理報告　手術日期：**110/03/12**　病理號：**S1008380**

  診斷醫師:吳鈺瑩 報告日期:110/03/24
  Pathologic diagnosis:
  1. Sigmoid colon, laparoscopic anterior resection --- Tubulovillous adenoma with focal high grade dysplasia. The bilateral cut ends and mesocolic lymph nodes (0/10) are free of tumor.
  2. IMA root, dissection --- Unremarkable fibroadipose tissue.
  3. Colon, cut end, distal, resection --- Unremarkable.
  Ancillary study for diagnosis:
  1. Step cut x 3 done for section E and F.
  2. IHC stain for AE1/AE3 (no abnormal infiltration) and desmin (no definite disruption) done for section E.
  Gross description:
  The specimen consists of 1) a segment of colon, 13 cm in length and 6 cm in proximal cut end circumference, 6 cm in distal circumference, and mesocolic soft tissue. A gray tan friable polypoid tumor, 3x3x2.5 cm, is noted, 2 cm to the distal cut end. On cross section, the tumor invades to the submucosa. There are metal staples at the root of tumor. The both cut ends are free of tumor grossly. 2) IMA root, a piece of tan yellow soft tissue, 4x2x1 cm. 3) distal cut end, a piece of tan mucosa-covered soft tissue, 2.5x2x0.7 cm. Representative part for section: A) specimen 2 B) specimen 3 C) proximal cut end D) vascular cut end E-F) polyp G) mucosa near tumor root H-S) mesocolic soft tissue and lymph nodes T-U) all the residual polyp.
  Note:
  The diagnosis is concurred in intradepartment consensus meeting on 2021/03/24.
  #T-59471_2 #M-82632_2 9 1105 00CM00
  #T-C4560_2 #M-09183_2 0 1105 000000

其它

- 隔離註記:接觸防護【CRE(檢)(20210324)】

出院時情況

- 15.計劃性出院

出院指示

藥名	劑量	劑量單位	用法	頻次	數量	數量單位	現況	天數	單位
AtenoloL 50mg tab	1	TAB	PO	QD	14	TAB	TH	14	天
(口)Takepron OD tab 30mg	1	TAB	PO	QDAC	14	TAB	TH	14	天
Estazolam tab 2mg	0.5	TAB	PO	HS	7	TAB	TH	14	天
Ultracet tab	1	TAB	PO	Q8H	21	TAB	TH	7	天
Duphalac syr. 300mL	20	ML	PO	QDPRN	1	ML	TH	14	天
Erymycin cap 250mg	1	CAP	PO	BID	28	CAP	TH	14	天
Through tab	1	TAB	PO	QN	14	TAB	TH	14	天

出院計劃

主治醫師 2062F 蔣鋒帆　/　住院醫師 2325G 劉仕楷

正式摘要(電子簽章：000000213343844)

W55 / W55

doctorRank:B
